# Relation between finger cold-induced vasodilation and rewarming speed after cold exposure

**DOI:** 10.1007/s00421-018-4012-y

**Published:** 2018-10-16

**Authors:** C. F. Kingma, I. I. Hofman, H. A. M. Daanen

**Affiliations:** Department of Human Movement Sciences, Faculty of Behavioural and Movement Sciences, Vrije Universiteit Amsterdam, Amsterdam Movement Sciences, Van der Boechorststraat 7, 1081BT Amsterdam, The Netherlands

**Keywords:** CIVD, Finger cold water immersion, Resistance index of frostbite, Cold injury risk

## Abstract

**Purpose:**

The risk for local cold injuries has been linked to poor cold-induced vasodilation (CIVD) during cold exposure and to poor rewarming after cold exposure. The purpose of this study is to establish the relation between CIVD and rewarming speed.

**Methods:**

Twelve participants immersed one hand in ice water for 30 min to evoke CIVD and the other hand in ice water for 10 min to investigate the rewarming profile. The ring, middle and index fingertip temperatures were monitored during hand immersion and the resistance index of frostbite (RIF) was calculated. RIF depends on minimal (*T*_min_) and mean (*T*_mean_) finger skin temperature and onset time. Rewarming was quantified using an infrared imaging system and the rewarming speed over 19 min was determined.

**Results:**

*T*_min_ (5.8 ± 3.0 °C) and *T*_mean_ (10.4 ± 3.0 °C) caused non-distinctive contributions to the total RIF-scores so that onset time (12.7 ± 3.1 min) became the dominant factor. A significant negative correlation between RIF and rewarming speed was found (*r*_*s*_ = − 0.60, *p* = 0.041).

**Conclusions:**

The negative relation between RIF and rewarming speed may be explained by the common observation that onset time relates to the temperature of fingertip tissue, while *T*_min_, *T*_mean_ and rewarming speed relates to body thermal status. The rewarming test is to be preferred over the CIVD test in terms of ease of use, but the predictive value of the rewarming test for cold injuries is limited, cannot replace the RIF since onset time of finger vasodilation is not included and should be further investigated.

## Introduction

Low ambient temperatures in combination with reduced body core temperatures may lead to reduced blood flow to the extremities in an attempt to preserve body heat (Daanen et al. 1997). Eventually, cold injuries may occur (Castellani and Young 2016), in particular in people that are physically active in cold environments (Cappaert et al. 2008). Cold injuries are debilitating (Carlsson et al. 2014, 2016) and occur during work-related pursuits such as in military personnel, but also in athletes such as runners, cyclists, and mountaineers. In mountaineers it is reported that cold injuries (hypothermia and frostbite) cover 3–5% of all injuries, whereas in Nordic skiers this is one-fifth of all injuries (Cappaert et al. 2008). In military personnel this varies from 0.2 to 366 per 1000 exposures (Cappaert et al. 2008). Cold injuries range from hypothermia to local cold injuries that can be of the freezing and non-freezing type (Long III et al. 2005).

The risk of cold injuries is lower when a good cold-induced vasodilation (CIVD) response is present. Wilson and Goldman observed that frostbite was absent when CIVD occurred (Wilson and Goldman 1970) similar to earlier observations of Iida (Iida 1949). CIVD is a paradoxical vasodilation that occurs predominantly in the fingers and toes after about 5–10 min of local cold exposure (Daanen 2003). This leads to an increase in the peripheral blood flow and thereby an increase in the local skin temperature. Subsequently, there are phases of vasoconstriction alternated with vasodilation, called the Hunting response (Daanen 2003). This peripheral vasoconstriction and vasodilation occurs in arterio-venous anastomoses (AVAs). These blood vessels are important for temperature regulation and function under control of the sympathetic nerve system. It has been shown that these AVAs have an important role in the CIVD response (Bergersen et al. 1999).

Daanen and Van der Struijs (2005) showed that the CIVD response is related to cold injury risk in marines. On the basis of the CIVD response they calculated the “Resistance Index of Frostbite” (RIF) according to the method of Yoshimura and Iida (1950). 12 Marines with cold injuries had a significantly lower RIF of 5.3 ± 1.6, as compared to 7.1 ± 1.6 for the remaining 198 marines. Thus, RIF might be related to a higher probability of having cold injuries. As part of the RIF, the amplitude and onset time of the CIVD response are valuable characteristics of the CIVD response since decreased amplitude and delayed onset of the CIVD response both have a predictive value for cold injuries (Cheung 2015). Although the RIF, amplitude and onset time of the CIVD could be useful as screening tools to predict a higher risk for cold injuries, it is unpleasant and inconvenient to provoke this CIVD response since the immersion lasts 30 min and it is often a painful experience.

Another way of determining the risk of cold injuries is by investigating the rewarming after cold exposure. Ruijs et al. (2009) investigated rewarming patterns using infrared thermography. Infrared thermography enables non-contact determination of skin temperature, which depends on local skin blood flow. The results showed a rewarming pattern consisting of three different phases after immersion in a water bath of 14.5 °C for 5 min. The first phase is a slowly/passive rewarming phase and after about 2 min there is a fast/active rewarming phase. Then there is a final phase where the temperature of the fingers oscillates around baseline temperature. Brandström et al. (2008) used these rewarming patterns to divide subjects in different rewarming groups for determining the risk for cold injuries. Cold injury occurrence during training was disproportionately higher in the slow rewarmers (4 of the 5 injuries) than the fast rewarmers (1 of 5). However, the experiment of Brandström et al. (2008) was performed with a small group of only 5 cold-injured subjects, thus with minimal statistical power. The rewarming test, however, is less unpleasant and inconvenient than provoking a CIVD response. Since rewarming profiles and a CIVD response both seem to have predictive value for cold injuries, it is, therefore, important to determine the relationship between a CIVD response and rewarming. Therefore, we performed an experiment in which one hand was used to provoke CIVD, while the other hand was immersed for only 10 min to investigate rewarming. Furthermore, we compared the rewarming profiles of the hand after the CIVD response and brief cold exposure in the other hand to examine the effect of immersion duration on rewarming. We hypothesized that the CIVD response quantified by the RIF in one hand was related to rewarming speed in the other hand.

## Methods

### Subjects

16 Subjects were recruited to participate in the study. None of the subjects had a history of cold injuries. Subjects with Raynaud’s syndrome were excluded from the test. All subjects were provided with information and instructions about the test and they gave their informed consent. Four subjects dropped out. Two of them did not complete the test due to dizziness and not feeling well. One subject was unable to maintain a stable hand position during video recording so that data could not be used for image processing. The other subject completed the CIVD measurement, but did not complete the rewarming after the CIVD response test due to too much pain during rewarming. Consequently, we used data of 12 subjects consisting of 7 women and 5 men with an age of 22.2 ± 2.4 year for analysis of CIVD response and rewarming profiles. The Scientific and Ethical Review Board of Vrije Universiteit Amsterdam approved the protocol.

### Procedure

Subjects were not allowed to smoke, eat, drink coffee and tea, be physically active or be exposed to cold for 2 h prior to testing. Furthermore, they were not allowed to drink alcohol 24 h prior to testing and they were asked to wear normal clothes consisting of underwear, trousers/jeans, shirt and/or sweater and shoes. When they arrived they accommodated to room temperature for 20 min while filling out a medical history form.

Thereafter, the subject was asked to place the palmar side of both hands on a cotton garment on the table. The infrared thermographic system (FLIR ONE for iOS, FLIR Systems AB, Täby, Sweden) with a sensitivity of 0.1 °C was placed on a tripod at ± 50 cm above the hands. A video recording of 20 s was made with a vertical view of the dorsal side of both hands to determine baseline temperature. Baseline temperature was defined as the temperature after 10 s recording.

Thereafter, 6 thermocouples (T-T-28M, Tempcontrol, Voorburg, The Netherlands) were attached to the palmar side of the distal phalanx of the ring, middle and index fingers of both hands using self-adhesive tape (DuoProtect, Dental & Cosmetic Care, Noord-Scharwoude, The Netherlands). To make sure the thermocouples were tightly connected, sports tape (Etos B.V., Zaandam, The Netherlands) was used to attach the sensors to the palm of the hand. Then, to provide dry hands for the rewarming measurements, thin surgical nitrile gloves were put on both hands. A plastic container was filled with ice cubes and water (0 °C). One thermocouple was hanging in the container and another was connected to a tripod to measure water temperature and room temperature respectively. The subjects placed both hands up to the metacarpal bones in the container filled with ice water, which was at the level of the heart. Water was mixed every 2 min with a plastic spoon to minimize thermal gradients within the container.

### CIVD

One hand [non-dominant hand (n = 6) and dominant hand (*n* = 6)] was immersed in the container with ice water for 30 min. Every 20 s the skin temperature of the fingertips was measured and data was stored in a laptop. Every 2 min subjects were asked to rate the pain they perceived in the immersed hand(s) on the Borg Scale, where 1 is no pain at all and 10 is extremely painful (Borg 1988).

Yoshimura and Iida devised a method to estimate the RIF from the temperature reaction to cold (Yoshimura and Iida 1950). In this experiment we calculated the RIF by taking the average of the RIF-scores of the three fingertips. The three variables used to calculate the RIF are: the lowest temperature after immersion of the finger before the initiation of the CIVD response (*T*_min_); the time at which the CIVD response initiates (Onset time); and the mean finger skin temperature during immersion from 5 min up to 30 min (*T*_mean_) (Yoshimura and Iida 1950). We used the same scoring system as Yoshimura and Iida to determine the RIF (Table 1). The RIF was determined by adding the points of the three characteristics together for each individual. The RIF varies from 3 to 9, whereby 3 is the lowest score which indicates a weak reaction to cold, and 9 is the highest score which indicates a strong reaction to cold. Two experimenters independently determined the onset time from the temperature chart. When values differed this was discussed until consensus was reached. The same procedure was followed for determining the highest temperature after immersion of the finger after CIVD response (*T*_max_). CIVD amplitude is defined as the difference between *T*_max_ and *T*_min_.

**Table 1:** Scoring system of Yoshimura and Iida to determine the resistance index of Frostbite (RIF)

Number of points	1	2	3
*T*_min_ (°C)	< 1.5	1.6–4.0	> 4.1
Onset time (min)	> 12	8–11	< 7
*T*_mean_ (°C)	< 4.0	4.1–7.0	> 7.1

The three variables to determine the RIF are: Lowest temperature after immersion of the finger before CIVD response (*T*_min_), initiation CIVD response (Onset time), mean finger skin temperature (*T*_mean_). The number of points for each parameter are added and yield a RIF-score ranging from 3 to 9.

### Rewarming

During the CIVD reaction, which was measured in one hand, the other hand [non-dominant hand (*n* = 6) and dominant hand (*n* = 6)] was only immersed for 10 min and was then taken out of the water. The glove was removed and the hand was placed with the palmar side on the cotton garment. The time between removal of the hands from the water bath and thermographic recording was less than 40 s. The first finger skin temperature measurement is Tfi,_0_. Then a time-lapse video of the cold recovery was made for 19 min with an interval of 8 s. Tfi,_19_ is the last finger skin temperature measurement.

After 30 min, the hand in which the CIVD response was determined was taken out of the water. The glove was removed and the hand was placed next to the other hand with the palmar side on the cotton garment. A time-lapse video of the cold recovery was made for 19 min with an interval of 8 s.

Vernier Thermal Analysis Plus for FLIR ONE was used for image processing of six regions of each hand. On each of the three fingers of the hands, a spot was selected on the dorsal fingertip and finger base. Temperature data from the selected spots was exported to Vernier Graphical Analysis 3.2 and then to Microsoft Excel 2011 (Microsoft Corporation, Redmond, WA).

### Thermographic variables and rewarming classification

The mean of index-, middle- and ring fingertip temperatures (Tfi) was determined for the following five points in time: baseline temperature, the end of 10 min cooling, and after 5 min, 10 min and 19 min of cold recovery, similar to Brändström et al. (2008). Even though water immersion duration and water temperature differed between their and our study, we used their criteria to make a subdivision in normal, moderate or slow rewarmers, which corresponds to the subdivision in the study by Dupuis (1987). This classification is based on the percentage of the 19-min rewarming period while vasodilation of the fingertips was present (%VD), which was the case when fingertip temperature was at least 0.1 °C greater than finger base temperature (Anderson et al. 2007; Schuhfried et al. 2000). Rewarming categories include: normal rewarming, Tfi_,19_ > 20 °C and %_VD_ > 50%; moderate rewarming, either Tfi_,19_ > 20 °C and %_VD_ ≤ 50%, or Tfi_,19_ < 20 °C and %_VD_ ≥ 5%; and slow rewarming, Tfi_,19_ < 20 °C and %_VD_ < 5%. In addition to the classification of rewarming groups, rewarming amplitude (Δ*T*_REWARMING_) is also measured. Δ*T*_REWARMING_ equals the temperature difference between the beginning (Tfi_,0_) and the end (Tfi_,19_) of the rewarming period. Since Δ*T*_REWARMING_ is measured over a period of 19 min in all subjects, it is an indicator of rewarming speed (°C/min). This procedure is followed for both Δ*T*_REWARMING_ after short immersion and Δ*T*_REWARMING_ after the CIVD response.

### Data analysis

Spearman correlation analysis was used to determine the relation between the CIVD response and rewarming response after brief cold exposure. To determine whether there are any differences between the rewarming after brief cold exposure and the rewarming after a CIVD response a paired sample *t* test was used. Finally, the relation between the RIF-score and Borg ratings of perceived pain was analyzed using Spearman correlation analysis. All analyses were performed using SPSS (SPSS Inc.^®^, version 25, Chicago, IL). Data are presented as mean ± SD with significance level set at *p* < 0.05.

## Results

### CIVD response

**Fig. 1:**
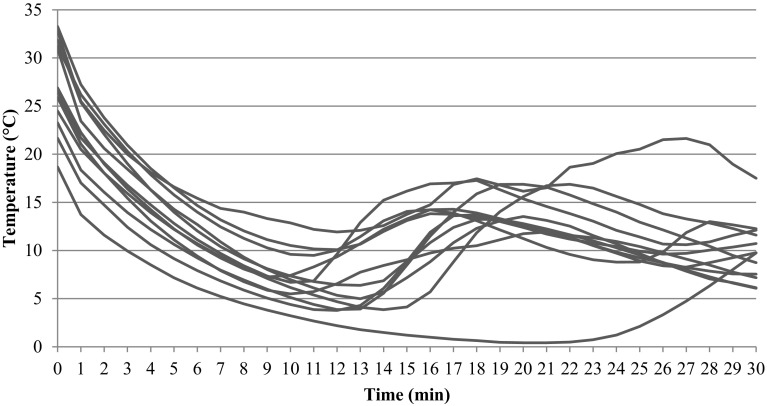
CIVD responses of all subjects (*n* = 12) during fingertip immersion in ice water for 30 min. All subjects show a drop in temperature after immersion and subsequently a rise in temperature

The CIVD response of the 12 participating subjects is presented in Fig. 1 and Table 2. Directly after immersion at t = 0 min, fingertip temperature dropped. Onset time averaged 12.7 ± 3.1 min. The average water and room temperature of all CIVD measurements were − 0.4 ± 0.1 °C and 23.8 ± 1.0 °C respectively. The mean RIF-score of the subjects was 6.8 ± 1.5.

**Table 2:** Results for *T*_min_, onset time and *T*_mean_ with corresponding RIF-scores and total RIF-score for the CIVD test; start and end temperature of the rewarming period and rewarming speed for the rewarming test and CIVD test

Subject	Sex	*T*_min_ (°C)	RIF-*T*_min_	Onset time (min)	RIF-onset time	*T*_mean_ (°C)	RIF-*T*_mean_	RIF-total	Trew, 0 (°C)	Trew, 19 (°C)	Rew speed (°C/min)	Trew, 0 CIVD (°C)	Trew, 19 CIVD (°C)	Rew speed CIVD (°C/min)
1	F	3.7	2	12.7	1	10.5	3	6	11.6	35.3	1.2	15.7	35.9	1.1
2	F	0.4	1	21.3	1	3.1	1	3	10.4	26.1	0.8	21.3	28.4	0.4
3	M	9.2	3	11	2	11.7	3	8	17.6	34.4	0.9	19.0	32.2	0.7
4	M	6.4	3	12.7	1	10.5	3	7	13.7	34.6	1.1	14.7	35.0	1.1
5	F	6.5	3	9.7	2	10.5	3	8	17.9	32.1	0.7	16.9	28.9	0.6
6	F	4.3	3	14.7	1	9.4	3	7	1.8	24.0	1.2	7.8	22.7	0.8
7	F	3.8	2	11.7	1	8.5	3	6	11.1	34.1	1.2	10.5	25.3	0.8
8	M	8.7	3	11	2	11.6	3	8	22.7	34.6	0.6	18.8	34.9	0.8
9	M	11.5	3	12.3	1	16.4	3	7	21.6	36.4	0.8	14.1	26.1	0.6
10	F	6.6	3	10.3	2	12.3	3	8	16.6	34.4	0.9	19.6	33.2	0.7
11	F	3.7	2	14.3	1	10.6	3	6	11.5	32.2	1.1	18.3	32.7	0.8
12	M	5.1	3	10.7	2	9.4	3	8	11.1	25.8	0.8	15.1	24.7	0.5
Mean		5.8	2.6	12.7	1.4	10.4	2.8	6.8	14.0	32.0	0.9	16.0	30.0	0.7
SD		3.0	0.7	3.1	0.5	3.0	0.6	1.5	5.7	4.2	0.2	3.9	4.5	0.2

Mean and standard deviation (SD) are shown in the two lower rows

*Rew* rewarming, *0* start minute of rewarming, *19* end minute of rewarming

### Rewarming

The subjects were categorized into normal (*n* = 8), moderate (*n* = 4) and slow (*n* = 0) rewarmers. Rewarming amplitude (Δ*T*_REWARMING_) was 18.1 ± 4.0 °C.

### CIVD and rewarming

**Fig. 2:**
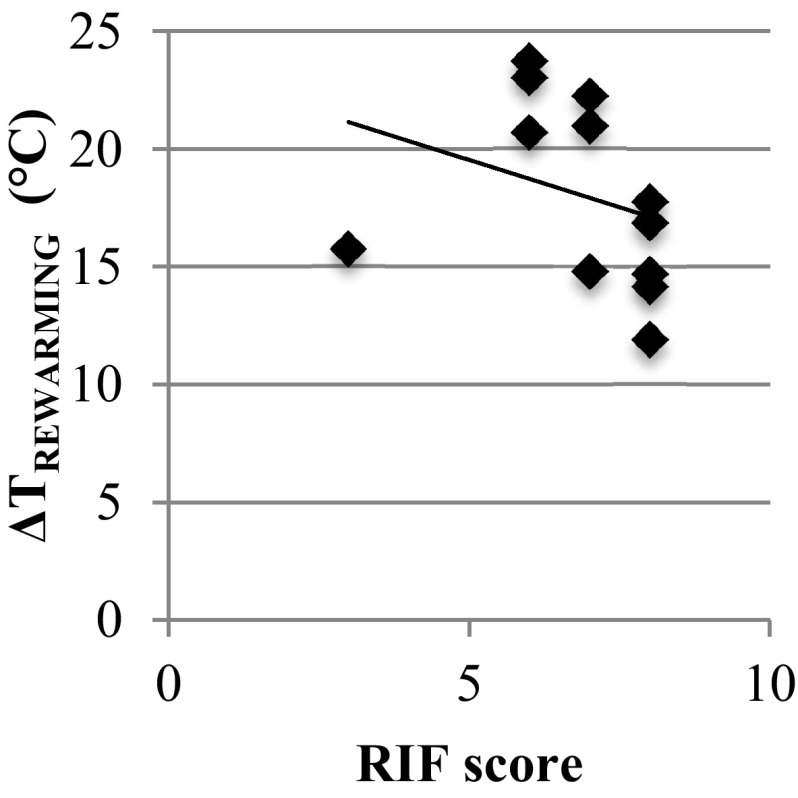
Relationship between Δ*T*_REWARMING_ (°C) and RIF-scores (*n* = 12)

A significant negative correlation between RIF and Δ*T*_REWARMING_ (*r*_*s*_ = − 0.60, *p* = 0.041) (Fig. 2) was found. No significant correlation was found between the RIF and rewarming category (normal/moderate) after brief cold exposure (*r*_*s*_ = − 0.05, *p* = 0.87). The Spearman correlation matrix for all variables is shown in Table 3.

**Table 3:** Spearman correlation matrix of onset time (min), minimal finger skin temperature (*T*_min_ in °C), maximal finger skin temperature (*T*_max_ in °C), amplitude (°C), mean finger skin temperature (*T*_mean_ in °C), RIF-score (range 3–9), rewarming speed (°C/min), finger skin temperature at the start of rewarming (Tfi, 0 in °C), finger skin temperature at the end of rewarming (Tfi, 19 in °C), and pain rating

	Mean	SD	Onset time (min)	*T*_min_ (°C)	*T*_max_ (°C)	Amplitude (°C)	*T*_mean_ (°C)	RIF-score	Rew speed (°C/min)	Tfi, 0 (°C)	Tfi, 19 (°C)	Pain score
Onset time (min)	12.70	3.11	1.00	− **0.62**	− 0.25	0.34	− 0.39	− **0.83**	0.51	− **0.59**	− 0.11	0.40
*T*_min_ (°C)	5.83	2.99	− **0.62**	1.00	0.44	− 0.46	**0.77**	**0.77**	− 0.52	**0.81**	0.47	− 0.35
*T*_max_ (°C)	14.76	3.05	− 0.25	0.44	1.00	0.50	**0.85**	0.19	− 0.04	**0.65**	**0.61**	− 0.40
Amplitude (°C)	8.93	2.69	0.34	− 0.46	0.50	1.00	0.07	− **0.61**	0.51	− 0.24	0.12	0.07
*T*_mean_ (°C)	10.38	3.04	− 0.39	**0.77**	**0.85**	0.07	1.00	0.49	− 0.27	**0.81**	**0.66**	− 0.48
RIF-score	6.83	1.47	− **0.83**	**0.77**	0.19	− **0.61**	0.49	1.00	− **0.60**	**0.60**	0.03	− 0.48
Rewarming speed (°C/min)	0.95	0.21	0.51	− 0.52	− 0.04	0.51	− 0.27	− **0.60**	1.00	− 0.57	0.04	− 0.06
Tfi,0 (°C)	13.95	5.73	− **0.59**	**0.81**	**0.65**	− 0.24	**0.81**	**0.60**	− 0.57	1.00	**0.70**	− 0.36
Tfi,19 (°C)	32.00	4.23	− 0.11	0.47	**0.61**	0.12	**0.66**	0.03	0.04	**0.70**	1.00	− 0.24
Pain score	3.59	1.72	0.40	− 0.35	− 0.40	0.07	− 0.48	− 0.48	− 0.06	− 0.36	− 0.24	1.00

Bold values indicate significance at *p* < 0.05 level

### Rewarming after brief cold exposure and rewarming after CIVD response

**Fig. 3:**
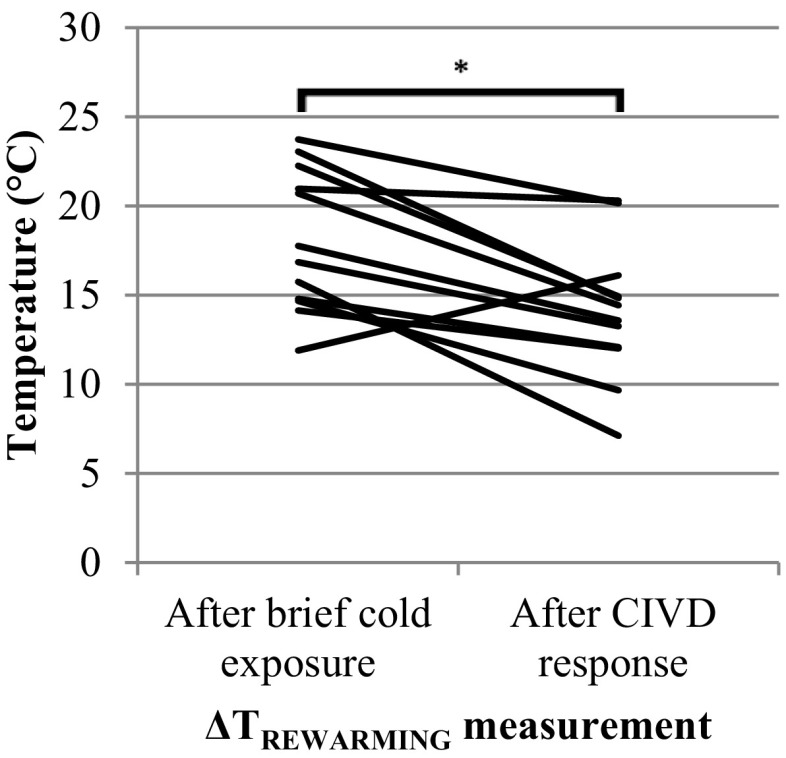
Temperature change of each subject (*n* = 12) during rewarming after 10-min cold exposure (Δ*T*_REWARMING_) and 30 min cold exposure (Δ*T*_CIVD_) (*p < 0.01)

A significant difference was observed between the temperature change during rewarming after brief cold exposure (18.1 ± 4.0 °C) and after the CIVD response (14.0 ± 3.8 °C) [*t*(11) = 3.89, *p* = 0.003] (Fig. 3). Corresponding rewarming speeds were 0.9 ± 0.2 and 0.7 ± 0.2 °C/min, respectively (Table 2).

### Pain rating

**Fig. 4:**
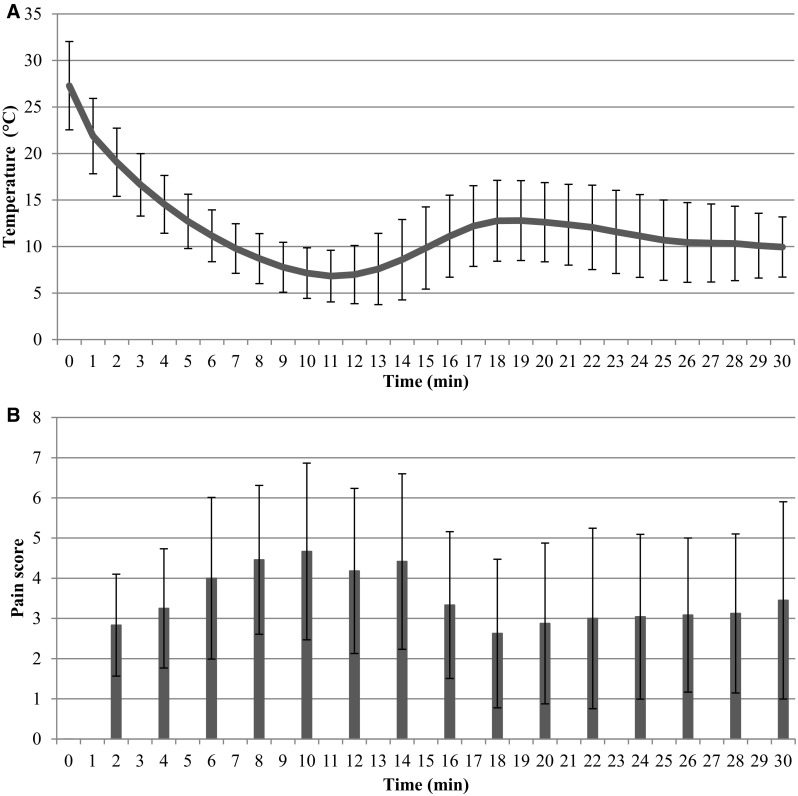
**a** Average fingertip temperature (mean ± SD) of all subjects (*n* = 12) during 30-min immersion in ice water. **b** Average pain score (mean ± SD) of all subjects (*n* = 12) during 30 min immersion in ice water

Average fingertip temperature was lowest at 11 min after immersion (Fig. 4a) and average pain score was maximal 10 min after immersion (Fig. 4b). The average pain score was negatively correlated with average fingertip temperature (*r*_*s*_ = − 0.84, *p* < 0.001).

## Discussion

Previous research has shown that both CIVD and rewarming have predictive value for the risk of local cold injuries, although the statistical power is low: only 12 subjects with cold injuries for CIVD (sensitivity 36%, specificity 92%) (Daanen and van der Struijs 2005) and 5 subjects with cold injuries for rewarming (Brändström et al. 2008). In the current study, we determined the relationship between the RIF, based on the onset time, *T*_min_ and *T*_mean_ of the CIVD response, and rewarming speed. We observed a small, but significant negative correlation between RIF and Δ*T*_REWARMING_ (*r*_*s*_ = − 0.596, *p* = 0.041). This means that subjects with a poor CIVD response in one hand, i.e., a low RIF, show fast rewarming in the other hand. Thus, the hypothesis that the CIVD response quantified by the RIF in one hand was positively related to rewarming speed in the other hand has to be rejected.

A possible explanation lies in the observation that the components *T*_min_, *T*_max_ and onset time did not equally contribute to the RIF-scores in our study. Table 2 shows that most subjects had relatively warm fingers, thus contributing 2.6 point to the RIF-score for *T*_min_ and 2.8 points for *T*_mean_. Only one subject had a *T*_mean_ score that was not equal to 3 (subject 2). As a result, the relative contribution of onset time becomes dominant in the RIF-score: 7 subjects scored 1 point, and 5 subjects scored 2 points. Thus, a RIF-subdivision was made in two groups: fast and slow CIVD. The theories about the onset of CIVD relate to the opening of arterio-venous anastomoses (AVA) in the skin of the fingertip due to a paralysis of the muscles in the AVA caused by the local low temperature (Daanen 2003). When the AVA’s are open, blood flow in the finger tissue depends on the alternating opening and closing of the precapillary sphincters in the arterioles that are under sympathetic control. This is why the body core temperature has such a large impact on peripheral blood flow in the cold (Daanen and Ducharme 1999; Daanen et al. 1997; Flouris et al. 2008). *T*_mean_ during immersion and rewarming speed are directly related to peripheral blood flow, while *T*_min_ is an indicator of the minimal blood flow during immersion. Thus, the three RIF-parameters onset time, *T*_mean_ and *T*_min_ are representative for different physiological functions. However, onset time, *T*_mean_ and *T*_min_ are not independent. When the body core is warm, for instance, the onset time is short and *T*_mean_ and *T*_min_ are elevated. In our study RIF is mainly dependent on onset time, while it can be argued that rewarming speed is mainly dependent on the thermal state of the body. Therefore, RIF and rewarming speed are not positively related. The mechanisms of onset of CIVD and blood flow through the finger tips are physiologically unrelated although it can be argued that a strong sympathetic drive may cause a faster cooling of the fingertip.

There is considerable variation in hand and finger temperature between subjects. Some subjects have mostly cold hands, others have warm hands. This is reflected in *T*_mean_ of the ‘CIVD-hand’ and in Tfi, 0 and to a lesser extent Tfi, 19 in the hand used for rewarming (Table 2). The large interindividual difference in cold/warm hands explains the high correlation between *T*_mean_ and Tfi_,0_ (*r* = 0.81—Table 3). *T*_mean_ is only one of the three constituents of RIF, and rewarming speed in the other hand is independent of the absolute values of Tfi_,0_ and Tfi_,19_ in the other hand (Table 3). Therefore, the positive correlation between *T*_mean_ and Tfi_,19_ is not contradictory to the negative correlation between RIF and rewarming speed.

Rewarming speed was higher after brief cold exposure (0.9°C/min) than after the 30 min immersion for determining CIVD (0.7 °C/min). This can be explained by the observation that the finger skin temperature of the hands directly after 30 min ice water immersion was 2 °C higher than after 10-min immersion (Table 2). This in turn was due to the CIVD reaction warming up the finger tips (see Fig. 1).

The phases of vasoconstriction and vasodilation are related to more pain and less pain, respectively (Cheung 2015; Kreh et al. 1984). The average fingertip temperature shows a minimum temperature after 11 min of immersion in ice water due to vasoconstriction and the maximum pain score is close to this moment (Fig. 4). After 11 min the fingertip temperature rises and later again a drop in temperature occurs, while pain score decreases and later increases. This leads to the negative relationship between fingertip temperature and pain score, which is in line with previous work (Cheung 2015; Kreh et al. 1984).

Although on average the worst pain was experienced after 10 min of hand immersion, some subjects with a long CIVD onset time experienced pain much longer than 10 min. In those cases the rewarming protocol is preferred over the CIVD test in terms of minimizing discomfort. The fact that two subjects had to be excluded from our experiment due to dizziness and not feeling well should be noticed. The subjects did not feel well during the first 10 min of the experiment while both hands were immersed in ice water. It is likely that the pain stimulus caused physical sensations that occur prior to a vasovagal syncope such as dizziness, sweating and a pale skin. The risk of having a vasovagal syncope should be taken into account while performing this experiment. Since these incidents occurred during the first 10 min, a syncope may occur during the rewarming test or CIVD test.

Our study has three main differences in the execution of the rewarming test compared to the study of Brändström et al. (2008). The first concerns the time of rewarming, which is 30 min in Brändström et al. (2008) and limited to 19 min in our study. This may lead to different %VD values, and therefore, to different rewarming category outcomes. In our study the categorization led to 8 normal and four moderate rewarmers, and we observed no significant relationship between the RIF-score and the rewarming category. The second difference is that we used ice water (−0.4 ± 0.1 °C) in our experiment. Daanen and van der Struijs (2005) evoked a CIVD response using a temperature of 0 °C but in the rewarming experiment of Brändström et al. (2008) the water temperature was 10 ± 0.5 °C. The higher water temperature may have affected the rewarming rate. We have selected ice water, since the temperature is easy to control and maintain and since ice water temperatures evoke good CIVD responses (Hirai et al. 1970). However, the optimal temperature for CIVD with minimal discomfort is about 8 °C (Mekjavic et al. 2013). Controlling water at higher water temperatures need the use of a thermostat bath. For a practical test for cold injury risk assessment, we opted for simple and reliable instrumentation. The third difference is that during hand rewarming in our study the other hand was in cold water, while in the study of Brändström et al. (2008) the other hand was exposed to air at room temperature. The advantage of combining the immersion of one hand with rewarming of the other is that the thermal status of the body core is similar and that comparison of CIVD and rewarming is not confounded by different body core temperature status. There may be some effect of the immersed hand on the non-immersed hand as indicated by Isii et al. (Isii et al. 2007). In their study the observed effect had a magnitude of about 1 °C which is minor compared to the changes observed using infrared thermography. In another study (Daanen 1997) seven subjects immersed one hand in 6 °C water, and the changes in the non-immersed hands were unrelated to the immersed hands. In summary, the effect of temperature fluctuations in the immersed hand on the non-immersed hand temperature fluctuations are minor, if present at all. Thus, rewarming may have been slightly slower in our study compared to the study of Brändström et al. (2008). This is an extra factor complicating the comparison of rewarming, next to the different water temperature and rewarming time.

The three differences in rewarming protocol (timing, water temperature and temperature of the non-rewarmed hand) may have caused differences in the rewarming rate, but apply to all investigated subjects and do not interfere with the observation that RIF is negatively related to rewarming rate and that there is no relation between rewarming category and RIF.

Since the hands were not pinned to the table during the rewarming measurement, the fingers could move outside of the vision cone of the infrared measuring system. Therefore, we moved the spot of the thermometer with this change of the hand, but this was insufficient for one subject, which we had to exclude from the study.

In conclusion, this study shows that the RIF test and rewarming test, both used for prediction of local cold injury risk, are inversely related and that no significant relation exists between rewarming category and RIF. Subjects with low RIF values, indicative for a high risk for local cold injuries, may have high rewarming rates, indicative for a low risk for local cold injuries. Conversely, subjects with high RIF values may have slow rewarming. The statistical power of the predictors for cold injury risk is rather poor; this applies in particular for the relation between rewarming speed and the risk for local cold injury that is based on only five subjects with cold injuries (Brändström et al. 2008). A weak point in the RIF predictor is that the test is based on CIVD in the fingers, while most cold injuries occurred in the feet (Daanen and van der Struijs 2005) that have different thermal control mechanisms than the hand (Cheung and Mekjavic 2007; Daanen et al. 2012; Maley et al. 2017; Norrbrand et al. 2017). More experimental data is required to make valid predictors for cold injury risk, in particular when aimed at the individual level. A promising avenue is to investigate the physiological mechanisms underlying inter-individual differences in cold injury risk and a closer look at the involvement of the sympathetic nerve system (Brändström et al. 2013).

## Abbreviations

AVA: Arterio-venous-anastomosis
CIVD: Cold-induced vasodilation
RIF: Risk index of frostbite
SD: Standard deviation
*T*: Temperature
Tfi: Finger skin temperature
